# Into the storm: the imbalance in the yin-yang immune response as the commonality of cytokine storm syndromes

**DOI:** 10.3389/fimmu.2024.1448201

**Published:** 2024-09-10

**Authors:** Amy Armstrong, Yuting Tang, Neelam Mukherjee, Nu Zhang, Gang Huang

**Affiliations:** ^1^ Department of Cell Systems and Anatomy, Long School of Medicine, University of Texas Health Science Center at San Antonio, San Antonio, TX, United States; ^2^ Department of Microbiology, Immunology, and Molecular Genetics, Long School of Medicine, University of Texas Health Science Center at San Antonio, San Antonio, TX, United States; ^3^ Division of Experimental Hematology and Cancer Biology, Cincinnati Children’s Hospital Medical Center, Cincinnati, OH, United States; ^4^ Department of Urology, Long School of Medicine, University of Texas Health Science Center at San Antonio, San Antonio, TX, United States; ^5^ Department of Pathology & Laboratory Medicine, Long School of Medicine, University of Texas Health Science Center at San Antonio, San Antonio, TX, United States

**Keywords:** cytokine storm syndromes, cytokines, hyperinflammation, immune response, yin-yang

## Abstract

There is a continuous cycle of activation and contraction in the immune response against pathogens and other threats to human health in life. This intrinsic yin-yang of the immune response ensures that inflammatory processes can be appropriately controlled once that threat has been resolved, preventing unnecessary tissue and organ damage. Various factors may contribute to a state of perpetual immune activation, leading to a failure to undergo immune contraction and development of cytokine storm syndromes. A literature review was performed to consider how the trajectory of the immune response in certain individuals leads to cytokine storm, hyperinflammation, and multiorgan damage seen in cytokine storm syndromes. The goal of this review is to evaluate how underlying factors contribute to cytokine storm syndromes, as well as the symptomatology, pathology, and long-term implications of these conditions. Although the recognition of cytokine storm syndromes allows for universal treatment with steroids, this therapy shows limitations for symptom resolution and survival. By identifying cytokine storm syndromes as a continuum of disease, this will allow for a thorough evaluation of disease pathogenesis, consideration of targeted therapies, and eventual restoration of the balance in the yin-yang immune response.

## 1 Introduction

**Figure 1:**
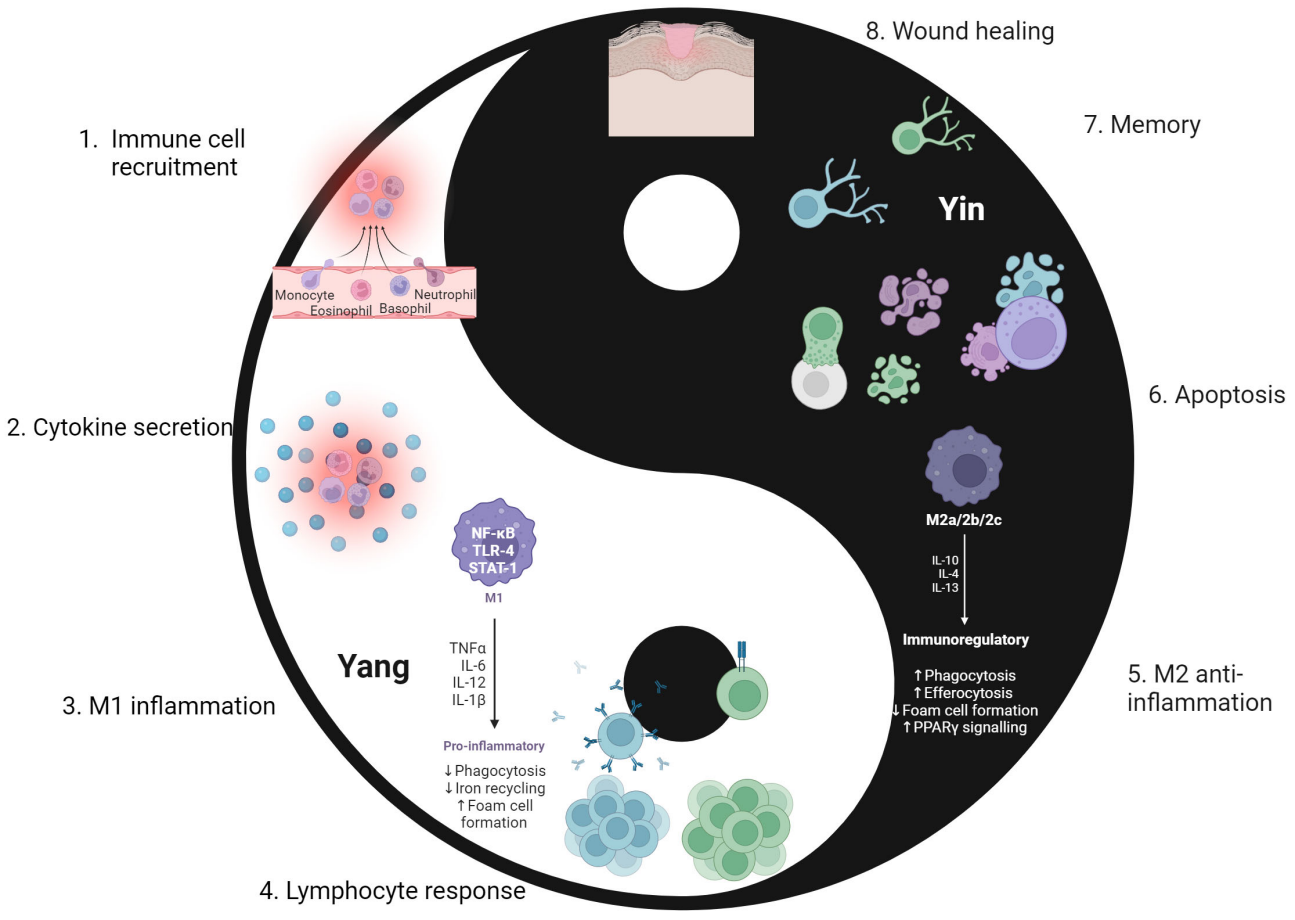
The cyclic “yin-yang” nature of the immune response. The immune response aligns with the yin-yang concept of complimentary forces. Yin represents a retractive or passive force, depicted as the immune contraction phase (5–8). Yang is the force that is active and expansive, as seen in the immune activation phase (1–4). Following repair of sites that were damaged by both pathogen and inflammatory processes, the immune system returns to a baseline and is poised to react to the next threat. 1. Upon encountering a pathogen, chemokine and cytokine gradients allow for recruitment of innate immune cells to the site of infection. 2. Innate immune cells secrete pro-inflammatory cytokines that cause further recruitment of immune cells, self and nearby immune cell activation, and the initiation of an inflammatory environment for further activation. 3. Stimulated from IFN-γ secretion from both self and other immune cells, macrophages differentiate into the pro-inflammatory M1 phenotype. This furthers the activation phase in the immune response. 4. Lymphocytes initiate the adaptive immune response, forming a more specific and targeted response to pathogens. This involves development, selection, activation, and differentiation into effector cells as lymphocytes enter the site of infection. 5. Successful elimination of the threat sets the stage for the contraction phase. Macrophages switch to the anti-inflammatory M2 phenotype, aiming to downregulate inflammatory processes from the activated phase. 6. Apoptosis of immune cells occurs, eliminating the excessive cells that were specifically recruited and activated for pathogen defense. This ensures the inflammatory and cytotoxic effector function of immune cells do not continue to persist. 7. Selected B and T lymphocytes undergo a complex process of differentiation into memory cells. Such memory lymphocytes residing in tissues or circulation for extended periods of time to serve as a record of past infection. This will allow for a faster and more robust response if the pathogen is encountered again. 8. Wound healing also occurs in steps 5-7, but its completion marks the end of the immune contraction phase. Following repair of sites that were damaged by both pathogen and inflammatory processes, the immune system returns to a baseline and is poised to react to the next threat. Created with BioRender.com.

The immune system must maintain a status quo through a balance of activation and contraction. While its main purpose is to mount a defense against external and internal threats to the host, it must also ensure that it does not cause excessive or unnecessary damage to the host in the process. A period of immune contraction is equally important to that of activation, allowing the immune response and body to return the baseline – status quo – state once the threat has been resolved (1–5). This complimentary and cyclic nature of the immune response is somewhat like that of the yin-yang concept in traditional Chinese philosophy (6) to ensure that there is a robust response to threats whilst still maintaining an equilibrium. As depicted in **Figure 1**, this philosophy consists of opposing yet interrelated forces. “Yin” is associated with contraction, passivity, and coldness, while “Yang” relates to expansion, activation, and warmth. Yin-yang is observed in different aspects of life such as night and day, cardinal directions of north and south, and traditional ideals of femininity and masculinity. The host demonstrates the yin-yang philosophy through activation and contraction in a normal immune response. The “yang” component is viewed through the initial response to microbial or internal threats by immune cells activation and inflammation, while the “yin” component is demonstrated through wound healing, immune cell death, and immunological memory after the threat is resolved. Both forces must be present and active to make certain a balanced immune response occurs.

When the balance is tipped in favor of activation for a prolonged period of time, the immune response becomes dysregulated and favors strong inflammatory responses. The exact mechanism driving a dysregulated immune response is not fully understood, but it is thought to be mediated by an interplay of different components that include genetics, immune cell dysfunction, and excessive secretion of pro-inflammatory cytokines (7). Although an immune response is present in most healthy individuals, it is important to emphasize that its specific response to perceived threats is not synonymous in all. Recent studies have postulated on how differences in responsiveness to vaccines and disease development or progression can be attributed to various factors such as age, genetics, and biological sex (8–11). This is representative of the diversity in the immune baseline of different individuals. The development of hyperinflammatory conditions can be helpful to understand how differences in the immune response can lead to varying outcomes.

Chemical signaling through chemokines and cytokines is responsible for the recruitment and maintenance of immune cells in the innate and adaptive immune responses. Innate immune cells sense microbial antigens by pattern recognition proteins, which initiates signaling pathways that activate NF-κB transcription factor for mass production of the cytokines IL-1, IL-6, IL-8, and TNF-α (12). These classical pro-inflammatory cytokines ensure that the inflammatory immune response is maintained through activation of endothelial cells, release of cytokines and chemokines, and recruitment of immune cells. With over fifty members, chemokines play an important role in the innate response through directing immune cells to sites of infections by gradients and assisting innate immune cells with communication, persistence, and activation of lymphocytes in the adaptive immune response (13). Lymphocytes in the adaptive immune response are similarly reliant on cytokines for activation, recruitment, and maintenance, such as IL-2 signaling needed for T lymphocyte survival (14). Chemical signaling is an integral part of the yang component in the immune response, but cytokine storm syndromes (CSS) can occur when it becomes overpowering.

A cytokine storm can occur when the activation phase of the immune response persists far beyond what is beneficial to the host. This is characterized by an unprecedented flood of pro-inflammatory mediators, resulting in a hyperinflammatory state. The term cytokine storm was initially documented in the context of a dangerous and uncontrolled system-wide immune response to T-cell engaging, antibody-mediated drugs such as with the OKT3 immunosuppressant to prevent organ rejection (15), and TGN1412 for treatment of B cell lymphoma and rheumatoid arthritis (16). The presence of a cytokine storm has also been discussed on the background of hyperinflammatory conditions such as septic shock (17), graft-versus-host disease (18), and hemophagocytic lymphohistiocytosis (19). More recently, the association of a cytokine storm in severe COVID-19 manifestations has reinvigorated interest in understanding the mechanism and pathology underlying this hyperinflammatory immune response (20–22). Fajgenbaum and June have provided an extensive review of a cytokine storm to consolidate the shared features of systemic cytokine release and multiorgan failure seen in cytokine storm syndromes (CSS), as well as guidance on therapeutic interventions (23). It is continuously necessary to consider how cytokine storm propagates a damaging and uncontrolled hyperinflammatory response in these individuals. Evaluating CSS allows for a deeper understanding of how various factors can culminate into a perpetual state of activation and disease pathology. A brief overview of major CSS in adult and pediatric patients has been summarized in **Table 1**. This current review aims to focus on seven of the most clinically relevant cytokine storm-mediated conditions: sepsis and septic shock, drug reaction with eosinophilia and systemic symptoms (DRESS), primary hemophagocytic lymphohistiocytosis (pHLH), secondary HLH and macrophage activation syndrome (sHLH/MAS), graft-versus-host disease (GVHD), immune-related adverse events (irAEs), and cytokine release syndrome with immune effector cell associated neurotoxicity syndrome (CRS/ICANS). The purpose is to explore how differences in the immune baseline of certain individuals disrupts the yin-yang of the immune response, leading to similar patterns of immune dysregulation and CSS. This immune baseline describes the state at which the host is not contending with pathogens or malignancies yet is still able to rapidly mount an immune response if a threat arises. For hyperinflammatory conditions, the activation phase is too strong and leads to pathological effects. Furthermore, long-term effects, sequalae, and promising therapeutic approaches for CSS are also reviewed.

**Table 1:** Overview of major cytokine storm syndromes.

Condition	Age group	Brief description	Associated cytokines	Symptoms and clinical manifestations	References
***Castleman disease (CD)***	Adult	• Lymphadenitis that occurs due to excessive lymphoproliferation	• IL-6, IL-1β, TNF-α	• “B-cell associated symptoms” • Hypercytokinemia • Hypergammaglobulinemia • Abnormal cellular function, organomegaly	• 24–26
***Cytokine release syndrome and immune effector cell associated neurotoxicity syndrome (CRS/ICANS)***	Adult and pediatric	• Extreme immune reaction of cytokine storm and neurotoxic events following CAR-T cell therapy	• IL-6, IL-8, IL-10, and IFN-γ	• CRS symptoms include fever, headache, body aches, malaise • Neurological events relate to altered consciousness, motor and cognitive decline, and seizures	• 27, 30
***Drug reaction with eosinophilia and systemic symptoms (DRESS)***	Adult and pediatric	• Hyperinflammatory immune response of skin rash and eosinophilia that can result from a variety of drugs	• IL-5, IL-10, IL-18, IFN-γ, and TNF-α	• Fever, itching, malaise • Lymphadenopathy, abnormal leukocyte counts, liver and kidney damage, and gastrointestinal problems	• 31, 32
***Graft-versus-host disease (GVHD)***	Adult and pediatric	• Donor immune cells attack and initiate widespread recipient tissue damage following a stem cell transplant	• IL-1, TNF-α, IL-2, IL-6, IL-10, IL-12, and IL-18	• Initially presents as liver dysfunction, gastrointestinal problems, and skin rash • It can evolve into eye and mouth dryness, obstructive lung disease, and neurological damage	• 33–35
***Primary hemophagocytic lymphohistiocytosis (pHLH)***	Pediatric	• Abnormal expansion and proliferation of T cells and macrophages results in hypercytokinemia, hemophagocytosis, and multiorgan damage • Pathogenic gene variants	• IL-1, IL-6, IL-10, IL-18, IFN-γ, and TNF-α	• Cytopenias of two or more cell lines and hepatosplenomegaly • Hemophagocytosis • NK dysfunction	• 19, 36, 37
***Secondary hemophagocytic lymphohistiocytosis and macrophage activation syndrome*** ***(sHLH/MAS)***	Adult	• The form of HLH that is more associated with adults • There are multiple possible triggers for this condition that are not directly related to genetics such as pathogens or autoimmune rheumatic conditions	• IL-1, IL-2, IL-6, TNF-α, and IFN-γ,	• This presents similarly to primary hemophagocytic lymphohistiocytosis • Other symptoms can include skin rash, neurological manifestations, pulmonary and renal failure, or cardiac dysfunction	• 38–40
***Immune related adverse events*** ***(irAEs)***	Adult and pediatric	• A variety of autoimmune-like and multiorgan dysfunctions that can occur after usage of immune checkpoint inhibitors	• TNF-α, IL-1, IL-6, IL-12, IL-17, and IL-23	• Drug hypersensitivity of the skin, central and peripheral neurological manifestations, colitis, pneumonitis, and endocrinopathies	• 41–43
***Langerhans cell histiocytosis*** ***(LCH)***	Pediatric	• Abnormal proliferation of myeloid cells that are destined to be antigen-presenting cells • BRAFV600E gene variant has been strongly associated with some confirmed cases	• IL-1, IL-1β, IL-4, IL-8, GM-CSF, TNF-α,	• Widespread bone lesions from infiltrating Langerhans cells and immune cell activation • Skin problems, pulmonary dysfunction, liver and spleen problems, neurodegeneration	• 44–46
***Multisystem inflammation syndrome in children (MIS-C)***	Pediatric	• Named for the system-wide hyperinflammation and organ dysfunction seen in a small portion of pediatric patients that were infected with SARS-CoV-2 • Unique from adult patients	• IFN-γ, IL-6, IL-10, TNF-α, IL-17, and IL-18	• Pyrexia, gastrointestinal problems, and skin rash • Neurological, respiratory, and cardiovascular problems are akin to that of Kawaski disease	• 47–49
***Sepsis and septic shock***	Adult and pediatric	• Systemic and hyperinflammatory immune activation that occurs in response to a pathogen that has entered circulation	• TNF-α, IL-1β, IL-6, and IL-8	• Cardiac dysfunction, tissue hypoperfusion, and organ damage • Disruption in the coagulation system	• 50, 51

## 2 Sepsis and septic shock

**Figure 2:**
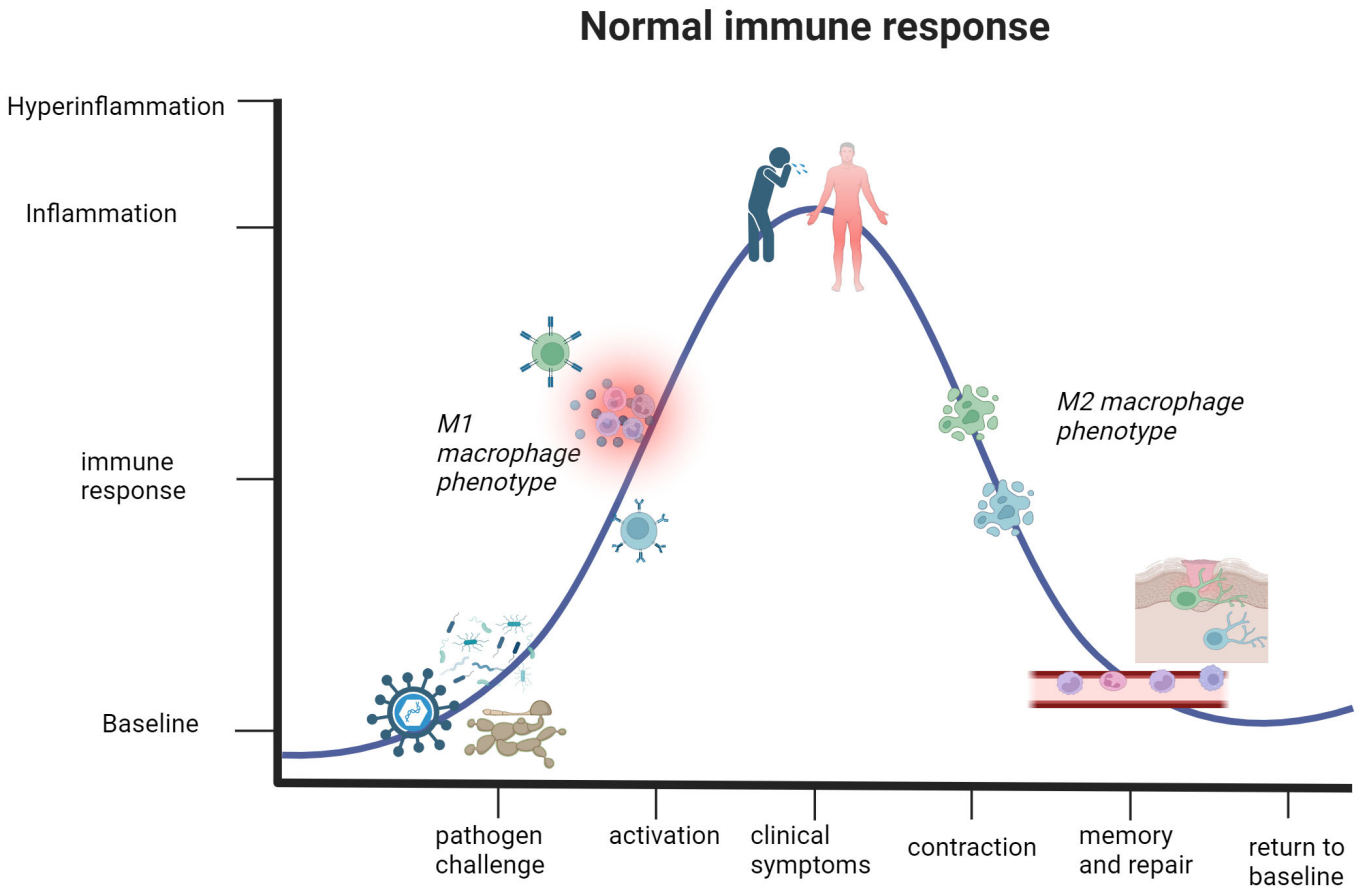
The immune response in healthy individuals undergoes a cycle of activation and contraction. This graph illustrates the trajectory of a normal immune response. Detection of a pathogen or threat to human health initiates immune activation and subsequent inflammation, partly thanks to M1 macrophages. The mounting immune response causes clinical presentation of symptoms in the host, but this activation phase will not reach a state of hyperinflammation that jeopardizes the safety and life of the host. Once the immune system eliminates the threat, the body enters the contraction phase defined by an anti-inflammatory environment and elimination of excess immune cells that are no longer necessary for active defense. Lastly, differentiation of quiescent memory cells and wound healing occurs. This represents a return to a baseline or steady state of being, which can respond to future threats. Created with BioRender.com.

By convention, sepsis and septic shock are described as an inappropriate system-wide response to a pathogen that has entered the bloodstream, involving excessive immune cell activation and subsequent secretion of pro-inflammatory mediators (52). As depicted in **Figure 2**, sepsis and septic shock begin as a normal immune response to a pathogen. When the pathogen enters the bloodstream, the immune response becomes overactive and may culminate into a septic condition. When the pathogen persists within the host, the secretion of classical pro-inflammatory cytokines also persists and leads to pathological consequences. The innate response is subject to widespread immune cell and complement activation for hyperinflammation (53). Persistence of microbial antigens in septic conditions also plays a role in the breakdown of the adaptive immune response. B and T lymphocytes present with an exhausted phenotype due to constant presentation with microbial antigens, leading to events of apoptosis and increased secretion of anti-inflammatory IL-10 (54). An overwhelming yang component of chemical signaling and immune cell activation leads to host tissue and organ damage in septic conditions.

**Figure 3:**
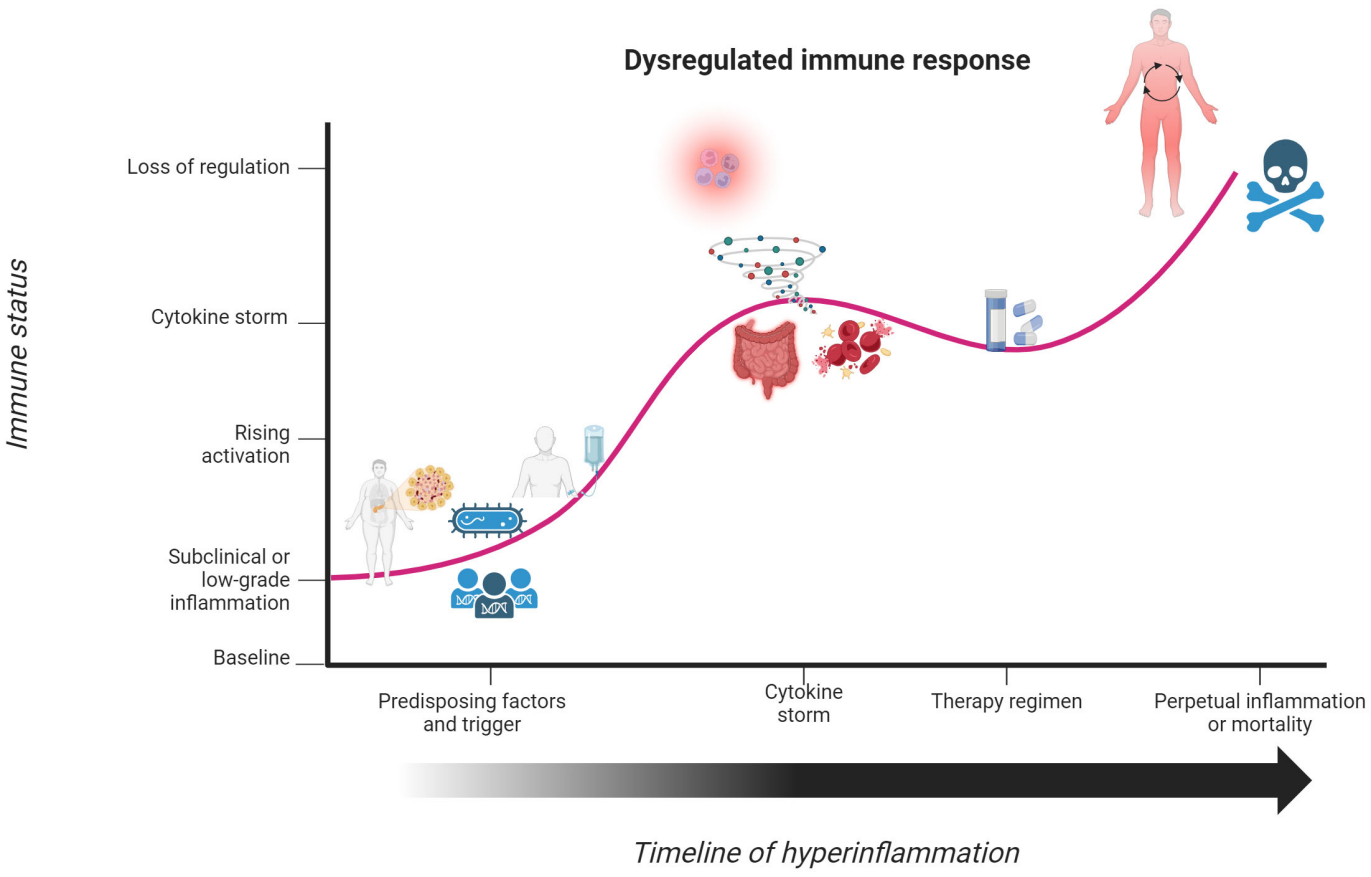
Underlying factors disrupt the yin-yang immune response and initiate a hyperinflammatory state. Individuals with underlying factors, such as chronic or pre-existing illnesses, cancer, or pathogenic gene variants, have an immune response that contrasts with that seen in **Figure 2**. In response to a perceived threat to the body or therapeutic trigger, individuals enter a state of immune activation to eliminate the threat. This immune response continues to mount until it culminates into a state of hyperinflammation or cytokine storm. Although steroids can temporarily stabilize the immune response, such effects do not fully correct the prolonged state of activation. This dysregulated activation phase of the immune response becomes so powerful and uncontrolled that patients enter a state of chronic hyperinflammation or meet their demise. Created with BioRender.com.

A study of ICU patients diagnosed with infection-associated septic shock reported a 70% overall positivity rate for one or more active microbe, further specifying that there were 47%, 62%, and 19% positivity rates for gram-positive bacteria, gram-negative bacteria, and various fungal species, respectively (50). The systemic activation of the immune response results in compromise of the endothelial cell barrier, leading to hypotension and multiorgan failure that defines septic shock (51, 55). Other clinical manifestations include cellular dysfunctions of disseminated intravascular coagulation and thrombocytopenia, tissue hypoperfusion and systemic vasodilation from cardiac dysfunction, and organ failure of the lungs, kidneys, and liver (56–61). Common symptoms of septic shock include pyrexia, elevated respiratory rate, tachycardia, and malaise (62). It should be reiterated that sepsis and septic shock represent the most extreme immune response to a pathogen, not the norm. The active inflammatory response in sepsis and septic shock coincides with the yang component of the immune response. As the onset and progression of septic shock are only discussed in ICU patients (52), this alludes towards an underlying factor contributing to susceptibility of this condition. Various comorbidities, such as diabetes, cancer, lung disease, and kidney or liver failure, are associated with the onset and overall severity in septic shock (63, 64). **Figures 2**, **3** illustrate how the immune response to a pathogen can lead to either successful clearance and immunological memory or persistent hyperinflammation in septic shock and related conditions, respectively. In **Table 1**, we also provide a brief overview of this condition, as well as other cytokine storm-related conditions. The existence of preexisting conditions could create a disturbed immune baseline that lowers the threshold required for immune activation, leading to difficulty in entering a state of immune contraction once the threat has ceased (**Figure 1**).

Given the potentially dire situation of sepsis and septic shock, there has been much interest in identifying biomarkers that can predict onset and severity in patients. C-reactive protein is elevated in this condition, but there is notable skepticism in using it as a biomarker because it is also elevated in many other conditions (58, 65). The rapid uptick of proinflammatory cytokines or cytokine storm has been more widely accepted and discussed as reliable biomarkers (66, 67). The cytokine profile of septic shock conditions was first detailed as elevations in TNF-α, IL-1β, IL-6, and IL-8 (68, 69). It has also been noted that elevations of IL-10 and CCL2 can be seen in certain patients with acute septic shock (70). A more in-depth discussion on the direct and indirect roles that various pro- and anti-inflammatory cytokines contribute towards septic shock progression has been described elsewhere (71). Although an elevation in proinflammatory cytokines is crucial for immune cell activation and response to a pathogen, septic shock patients are unable to control this activation phase. Corticosteroids are universally acknowledged as the standard of treatment for cytokine storm syndromes (CSS), but controversy remains on its true efficacy and sustainability in patient outcomes (72–75). This has been illustrated by the dismal reports of a 43.1% to 62% mortality rate within a 5-year follow-up period seen in three separate studies (76–78). Immunomodulatory drugs have been utilized as an alternative to steroids for sepsis and septic shock, but widespread approval and acceptance of this treatment regimen is far from complete (79, 80). For those that recover from septic shock, the dysregulated immune response is far from over. A strong yang component allows for hyperinflammation and organ damage seen in sepsis and sepsis shock, but some patients can develop a yin component that is equally detrimental. It is estimated that 33% of survivors may develop a chronic critical illness with a background of persistent inflammation, immunosuppression, and catabolism syndrome (PICS) (81). First proposed by physicians (82), PICS is characterized by recurrent infections due to a slew of opportunistic pathogens, wasting due to metabolic dysfunction, and low yet persistent levels of pro-inflammatory cytokines (83, 84). The damaging effects of septic shock can persist long after the illness has been treated, leaving the patient in an immunosuppressed state that directly opposes the hyperactive immune response from the cytokine storm. Yin and yang can be present in both the sequalae and main symptomatology of septic shock, but the bias towards either response is damaging. A combination of intrinsic factors and comorbid conditions position patients into a state of imbalance in the yin-yang immune response. From this point, a pathogenic trigger initiates a state of immune activation, leading to a cytokine storm and clinical manifestations of sepsis and septic shock (**Figure 3**).

## 3 Drug reaction with eosinophilia and systemic symptoms

The term “adverse drug reactions” was initially coined to describe individuals that developed skin rash and eosinophilia following usage of anticonvulsant medications (31). This condition is presently known as drug reaction with eosinophilia and systemic symptoms (DRESS), with reports expanding to include many commonly prescribed drugs and cancer immunotherapies (32, 85–88). The underlying immunopathology in DRESS is driven by components of both the innate and adaptive immune response. Helper T lymphocytes categorize drug antigens as foreign and launch a type 2 immune response to secrete IL-4, IL-5, and eotaxin (89, 90). These chemical signals then recruit eosinophils to sites of inflammation where they activate and release granules for further inflammation and tissue damage. It is also believed that latent viral reactivation in monocytes and macrophages are able to infect helper T cells and further contribute to the hyperinflammation seen in DRESS (90). The combined effects of drug hypersensitivity and viral reactivation lead to the cytokine storm and clinical manifestations seen in this condition.

Classical features of DRESS become apparent weeks to months after initial intake of the drug. These diverse symptoms include fever, skin eruptions, lymphadenopathy, and facial swelling (85). Histopathology of patients can reveal leukocytosis, mainly related to eosinophil infiltration into the skin (32). As described in **Table 1**, multiorgan manifestations are associated with this condition. Skin rash is one of the defining features of DRESS, characterized as having a maculopapular form and covering up to 50% of total body surface area (91, 92). Other clinical manifestations include pulmonary dysfunction, neurological problems such as speech and motor control deficiencies and altered levels of consciousness, liver failure, and acute kidney injury (89, 93–98). Organ manifestations in DRESS are reminiscent of those seen in other cytokine storm syndromes (CSS) (23). The low reports of this condition compared to the widespread and systemic usage of prescription drugs alludes to DRESS as an infrequent problem. While it is challenging to pinpoint the exact incidence of DRESS in the general population, it has been estimated to fall within the ranges of 1 in 1,000 to 1 in 100,000 (31). A study of electronic health records aligns with the lower range of incidence, reporting a prevalence of 2.18 out of 100,000 (98). The hyperinflammatory immune response to drugs and subsequent pathological consequences represent a strong bias towards the yang component of the immune response, directly contrasting with the balance illustrated in **Figure 1**. Elevated cytokines in DRESS further reveals its similarities to other CSS.

It has been postulated that reactivation of latent viruses, such as human herpesvirus 6 (HHV-6), Epstein-Barr virus (EBV), and cytomegalovirus (CMV), can overstimulate immune cells and lead to a cytokine storm, but is unclear whether viral reactivation is a mediator or consequence of DRESS (91, 99). A comparative analysis of patients diagnosed with COVID-19, COVID-19 with concurrent maculopapular rash, or DRESS found that there were similar elevations of CXCL9, CXCL10, CXCL11, IFN-γ, IL-10, TNF-α, and IL-18 across the three groups (100). There is a growing interest on how cytokine storm-like manifestations to different drugs can perpetuate the hyperinflammatory response in (drug reaction with eosinophilia and systemic symptoms (DRESS). It has also been shown that some DRESS patients have pathogenic variants in the CYP2C9*3 gene, resulting in diminished ability to metabolize drugs (101–103). The presence of latent viral reactivation and genetic abnormalities represent a disturbed yin-yang immune baseline, allowing for an immune response to drugs that is both hyperresponsive and damaging. To dampen this immune activation phase, topical skin steroids or systemic corticosteroids are used as a first-line therapeutic (104, 105). Alternative treatments, such as intravenous immunoglobulin and cyclosporin (106–109), can be used but are not as widely accepted nor implemented as steroids. The hyperinflammatory immune response seen in this condition can persist far beyond therapeutic intervention and resolution of symptomatology, as demonstrated in **Figure 3**.

Although not reported in all patients, there has been linkage of DRESS resolution and autoimmune sequalae (87, 91, 92, 94). In a retrospective study of 55 DRESS/drug-induced hypersensitivity (DIHS) patients, nine patients generated autoantibodies nearly a decade before the onset of an autoimmune condition (110). Furthermore, the development of autoimmune conditions occurred in a sequential order within 7.5 years of the initial observation. The disturbed yin-yang immune response in these individuals reaches far past the presentation of DRESS, prolonging the dysregulated immune baseline through the development of autoimmunity. For these individuals, the inability of the yin response to occur leaves them in a state of persistent activation. While usage of steroids as a first-line therapeutic provides immediate relief for symptomatology, the delayed presentation of autoimmunity indicates that it is unsuccessful in correcting the underlying immune dysregulation from DRESS.

## 4 Primary hemophagocytic lymphohistiocytosis

Hemophagocytic lymphohistiocytosis (HLH) is a hematological malignancy defined by defective cytotoxic functioning of T and NK cells, leading to rapid expansion and activation of macrophages and lymphocytes for persistent inflammation through hypercytokinemia and hemophagocytosis (111, 112). Immunopathology of this condition demonstrates a complex interplay of immune cells and hyperinflammation. Overactivation of these cells is viewed as a compensatory mechanism for their inability to release perforin on and subsequently lyse infected or malignant target cells (113). The overabundance of cytokines such as IFN-γ leads to activation of macrophages, furthering the cytokine storm by the defective cytotoxic cells. Macrophages then become hyperactive and display the key feature of this condition through consumption of red blood cells (114). Dysfunction of multiple immune cell types is then seen in the widespread organ and tissue damage of HLH patients.

This condition is further characterized into having primary and secondary forms (115). A brief summary of pHLH has been provided in **Table 1**. Hypercytokinemia and rapid expansion of leukocytes in HLH aligns with the yang phase of the immune response. The primary HLH (pHLH) form is associated with pediatric patients, illustrated in two separate reports by which most clinical presentations and diagnoses occurred before the patient’s first birthday (36, 116). Commonly noted symptoms include fever, rash, jaundice, lethargy, persistent viral infections, and swollen lymph nodes. As discussed in the previous conditions, hyperinflammatory manifestations of pHLH are known to occur in a system-wide manner. Poor prognosis is associated with the 30-70% of patients that develop neurological manifestations related to altered levels of consciousness, focal issues, and seizures (117), reported by one retrospective study to be associated with a 39.1% mortality rate in pHLH patients (118). Additionally, another retrospective study found a strong linkage between liver failure and confirmed pHLH phenotype (119). The excessive immune cell activation and organ damage is characteristic of an overactive yang component of the immune response, as seen with septic shock and DRESS. One group reported a pHLH incidence of 1.2 cases per million children (120), signifying the rarity of this hyperinflammatory disorder. Similar to DRESS, the low incidence of this condition speaks to an intrinsic difference in the immune baseline of such children.

For pediatric patients, it is more relevant to focus on genetic or inborn errors as drivers of disease pathology. Compared to adults, pediatric cohorts have not experienced the passage of time that allows for exposure to environmental factors and culmination of genetic damage that drives adult-onset pathologies. The genes most commonly associated with primary hemophagocytic lymphohistiocytosis (pHLH) onset are divided into those that dysregulate cytotoxic functioning of T and NK cells (PRF1, UNC13D, and STX11), lead to the pigmentation disorders of Griscelli syndrome, Chediak-Higashi syndrome, and Hermansky-Pudlak syndrome (RAB27A, LYST and AP3B1), or interfere with inflammasome formation (XIAP, NLRC4, and CDC42) (121–123). Hypercytokinemia and pHLH are common end results of these gene defects, resulting from overactivation of CD8+ cells, NK cells, and macrophages that attempt to compensate for defective effector functions (37, 124, 125). As previously discussed, multiorgan damage and hyperinflammation ensue from these pathogenic gene variants. With regards to cytokine storm, both forms of HLH share the cytokine profile of elevated IL-1, IL-6, IL-10, IL-12, IL-18, IFN-γ, and TNF-α (125–127). Recent studies of HLH found that increased ratios of IL-10 to IFN-γ (128) or concurrent elevations in IL-10 and IL-13 (129) were associated with the pHLH form. Therapeutic options for pHLH patients also focus on controlling the cytokine storm. HLH-94 and HLH-2004 protocols provide guidance on controlling the system-wide inflammation, seen with first-line therapeutics of the corticosteroids prednisolone and methylprednisolone (130, 131).The chemotherapeutic etoposide is also described as a treatment option, but its usage is mainly concurrent with steroids (36, 132). A stem cell or bone marrow transplant could prove curative for pHLH, but it is not without its own set of risks. One retrospective study found that 39 out 61 pHLH patients that received a stem cell transplant died before the 5.5-year follow-up period, either due to graft failure or disease progression (133). Of 51 observable patients, some form of graft-versus-host disease (GVHD) occurred in 48% of them. The contraction or yin phase of the immune response forcibly induced by steroids only serves to temporarily placate the hyperinflammatory state of pHLH patients, leaving their stressed immune system open to attack by opportunistic pathogen. Even those cured of pHLH from a stem cell transplant are not free of a dysregulated yin-yang immune response as the threat of developing GVHD is ever-present.

## 5 Secondary hemophagocytic lymphohistiocytosis

**Figure 4:**
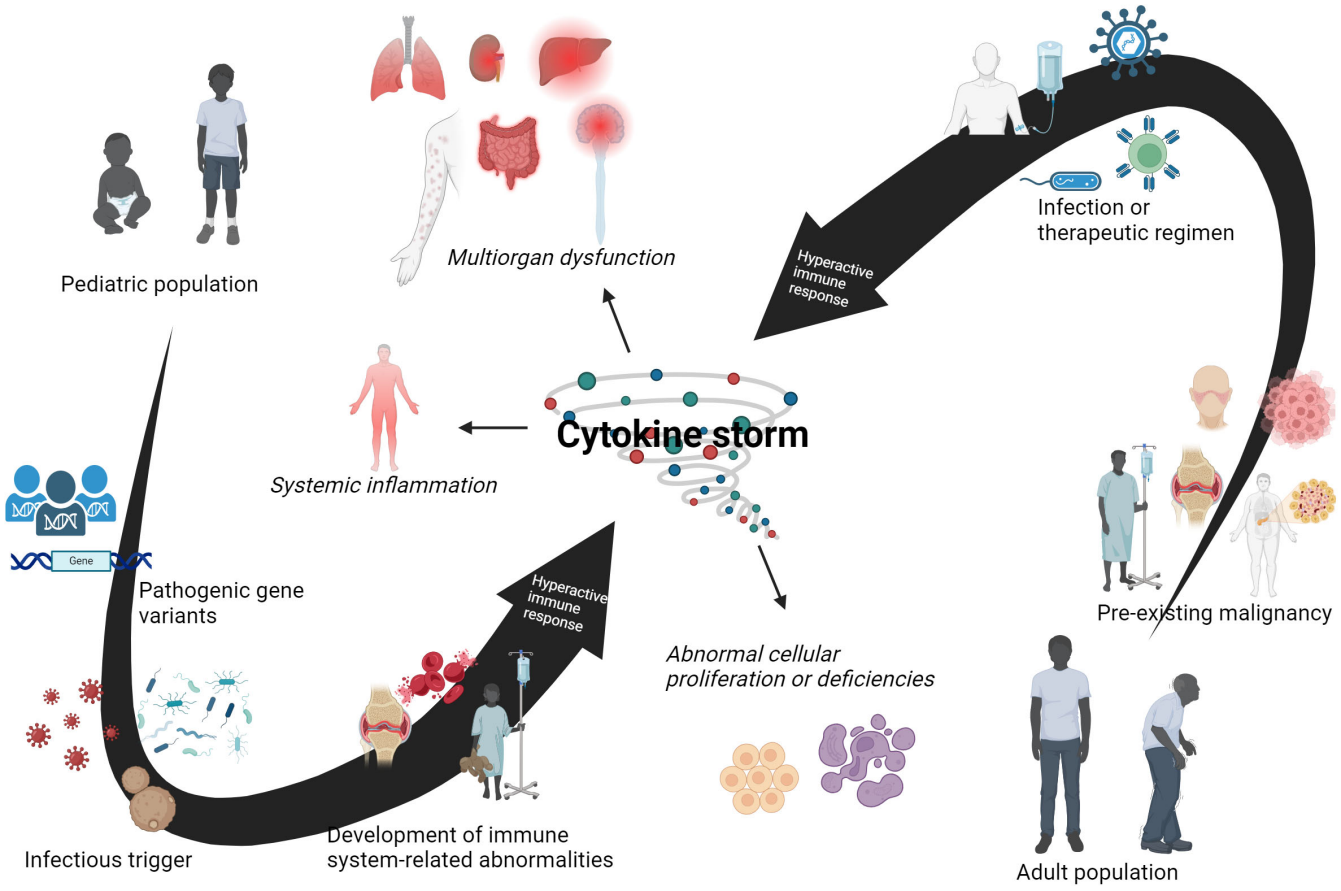
Different age-related factors contribute to cytokine storm syndromes (CSS). This is a schematic for intrinsic and extrinsic components in both pediatric and adult patients that can lead to the development of CSS. It is understood that different factors contribute to disease onset and progression in these two age groups. Pathogenic gene variants play a major role in a disturbed yin-yang immune baseline in pediatric patients, which is further exacerbated by subsequent encounters with infectious agents. Immune-related disorders can occur and culminate into CSS and related conditions in young patients. For adult populations, a pre-existing malignancy can be present before onset of CSS symptomatology. This imbalanced yin-yang immune response is hyperresponsive towards infectious or therapeutic triggers, leading to the development of a CSS in adult patients. However, there are exceptions to these observations. Pediatric patients can have a pre-existing illness that is not linked to genetic abnormalities. Adult patients with CSS have been identified to have pathogenic gene variants that may contribute to syndrome onset. An infectious trigger or therapeutic regimen may be enough for some adults to develop CSS. Nevertheless, both pediatric and adult patients are capable of developing CSS. Created with BioRender.com.

Genetic abnormalities were initially credited with driving hemophagocytic lymphohistiocytosis (HLH) pathogenesis, but the later discovery of HLH symptoms in children diagnosed with juvenile rheumatoid arthritis provided a new perspective into disease onset (134). Patients experience the same yang component and immunopathology seen in pHLH, with overactivated macrophages and T cells driving a hyperactive immune response. **Table 1** has provide an overview of this condition that will be further detailed in this section. Clinical features of fever, jaundice, skin rash, lymphadenopathy, and lethargy align with those described in pHLH. Termed as secondary HLH (sHLH), this form of HLH is characterized with having both an older age demographic and variety of triggers. A retrospective study of the US National Inpatient Sample database found a bimodal distribution of sHLH disease incidence in the age groups of 16-30 and 56-70 (135). This older age demographic is further illustrated by two separate studies by which the median age of sHLH diagnosis was reported to be over 50 years old (136, 137). Due to this older age demographic, non-genetic components are more implicated with disease when compared to pHLH. Rheumatic autoimmune conditions, such as systemic lupus erythematous (SLE), systemic idiopathic juvenile arthritis (sIJA), and adult-onset Still’s disease (AOSD), are commonly cited triggers for the macrophage activation syndrome (MAS) portion of sHLH (126, 138, 139). Other triggers of sHLH include pathogens like EBV and fungi, cancer, or treatment for hematological cancers, such as chimeric antigen receptor T (CAR T)-cell therapy (38, 39, 140). Underlying conditions or infections demonstrate an inclination towards an activated immune response, similar to that seen in the association of comorbidities with septic shock conditions. A simplified overview of different factors that contribute to cytokine storm syndromes (CSS) presentation in pediatric- or adult-associated conditions are summarized in **Figure 4**, as seen in pHLH and sHLH. Two separate genetic analyses of sHLH have also revealed a surprisingly large number of variants in pHLH-associated genes, including UNC13D, LYST, and PRF1 (141, 142). A variety of different factors, such as genetics and underlying conditions, can compound to initiate the cytokine storm and organ damage observed in sHLH.

The cytokine storm of IL-2, IL-6, TNF-α, and IFN-γ observed in both forms of hemophagocytic lymphohistiocytosis (HLH) induces a hyperactivated state of both macrophages and cytotoxic T cells. This positive feedback loop of cytokine secretion, immune cell activation, and multiorgan damage is similar to the effects seen in sepsis and septic shock conditions (40). Furthermore, there is also discussion of elevated IL-1 and IL-18 in the cytokine storm of HLH (143). To delineate the two forms of HLH, there have been concerted efforts to determine cytokine profiles that are exclusively predictive for either form. When compared to pHLH, one study found that a cytokine profile with elevated IL-4 and IFN-γ was predictive for sHLH patients (144). Another study compared non-HLH vs. HLH adult patients, reporting an elevation of familiar cytokines such as IL-10, IL-18, IFN-γ, and TNF-α, while also noting some rarely discussed chemokines such as eotaxin, IL-7, and MCP-1 (145). Tang and colleagues also discuss a seminal study by which a novel mouse model of elevated IL-10 and IL-18 produced an sHLH phenotype (146). This variety of elevated pro-inflammatory cytokines underlies the complexity in elucidating the cytokine storm and perpetual immune activation that occurs. The disturbance of the yin-yang immune response seen in **Figure 1** allows for an increased activation phase and cytokine storm development. Therapeutic guidelines follow that of pHLH by advising usage of etoposide and steroids as a broad attempt to quash the hyperinflammatory response (39, 117, 130–132). Rituximab for EBV-mediated sHLH and the anti-IL-1 cytokine therapy anakinra (147–150) have been explored as alternative options for treatment. These alternative therapeutic options aim to target specific mechanisms or components of the disease over induction of systemic immunosuppression through steroids. Although the field relies heavily on steroids to treat sHLH, statistics on sHLH survival rate do not speak to the efficacy of this treatment. When looking at ICU patients, one report found that 39% of sHLH patients died during the time of their stay (151) while a separate study estimated a 74% mortality rate at the 6-month follow-up (152). Given the high mortality rate of sHLH, this suggests that the yang immune response of patients is not complimented by the anti-inflammatory and retractive yin component. Patients still experience hyperinflammatory responses that persist throughout their lifetime, resulting in a complete breakdown of the yin-yang immune balance or mortality. The abysmal outcomes of sHLH indicate that novel therapeutic interventions are necessary to properly address the underlying hyperinflammation.

## 6 Graft-versus-host disease

In the mid-twentieth century, the term “secondary syndrome” was used to describe mice that died from multiorgan dysfunction following total-body irradiation and isologous transplantation of bone marrow cells from a donor mouse (153). This has since been renamed as graft-versus-host-disease (GVHD) and understood to occur due to donor T lymphocytes reacting to MHC molecules of the recipient, inducing a hyperinflammatory immune response and system-wide tissue damage (154, 155). The mismatch of human leukocyte antigen (HLA) is the main factor in driving GVHD onset. On a cellular level, donor T cells recognize both the self-peptides presented by recipient HLA class I and II molecules and polymorphic residues on HLA molecules themselves as foreign, thus initiating an inflammatory response (156). It is widely acknowledged that T lymphocytes in the adaptive immune response are responsible for initiating the cytokine storm and organ damage seen in GVHD. The yang or activation phase of the immune response is most prevalent in this condition. GVHD was originally categorized into acute (aGVHD) and chronic (cGVHD) forms depending on if main symptomatology occurred within 100 days of or post-100 days after a BMT (157–159). The field has since shifted towards a framework that uses the extent and severity of organ manifestations to typify this condition (160, 161). General features associated with GVHD onset include fever, rash and other skin eruptions, jaundice, diarrhea, nausea, and vomiting. Skin rash, gastrointestinal involvement, and liver dysfunction are considered to be some of the hallmarks of aGVHD onset (162–164), further expanding to reports of disturbed mucosal tissues of the eyes and mouth, dyspnea, and muscle aches in cGVHD (165–167). A concise summary of pathogenesis and clinical symptoms has been provided in **Table 1**. Acute and chronic forms of GVHD will not be considered separately due to the same common denominator of BMT as the initiator of pathology. As cytokine storm syndromes (CSS), GVHD and DRESS are similar in that onset is mediated by a hyperinflammatory reaction to therapeutic interventions intended to improve patients’ health. A bone marrow or stem cell transplant can be used to treat a variety of hematological disorders, autoimmune conditions, and inborn errors of metabolic function (168–170). Both malignancy-associated sHLH and GVHD have pre-existing conditions that serve as a predisposing factor for a hyperinflammatory immune response, although it is the treatment with a BMT that can specifically trigger GVHD. **Figure 3** illustrates how multiple factors can contribute to GVHD onset, as well as other CSS.

As a known cytokine storm syndrome (CSS), there has been interest in the cytokine profile responsible for graft-versus-host disease (GVHD) onset and severity. Conditioning regimens are necessary to prepare the recipient for engraftment, but their very nature may serve to further exacerbate the yin-yang immune dysregulation in patients. Immunosuppression and tissue damage can create an inflammatory environment by which donor lymphocytes are stimulated to expand and rapidly secrete cytokines (171, 172). Some of the earliest work on this unique profile found IL-1 and TNF-α as some of the main contributors of GVHD (35). More recent reports of the cytokine profile in GVHD include elevated levels of IL-2, IL-6, IL-10, and IL-17 (173, 174). Cytokine storm can play a central role in GVHD pathogenesis, highlighted by the difference in T-cell response and associated cytokine secretion in the two forms of this condition. The initial presence of Th1 cells secreting IL-12 IL-18, IFN-γ, and TNF-α in the inflammatory phase of aGVHD can switch to Th2 and Th17 cells secreting IL-4, IL-17, and IL-2 with cGVHD (174, 175). This can be viewed as the immune system trying to complement the hyperinflammation with the yin component, but ultimately failing to, resulting in the occurrence of cGVHD. For treatment options, the National Comprehensive Cancer Network (NCCN) unanimously recommends the use of either topical steroids or systemic corticosteroids depending on the extent and severity of tissue and organ involvement (176). However, there is concern for patients that become unresponsive or steroid-refractory (sr-GVHD) (177–182). For these cases, other treatment options include the FDA-approved JAK 1/2 inhibitor for sr-GVHD, as well as cellular products or antithymocyte globulin (178, 182). One study on the prognosis of aGVHD patients noted that more than 50% of all patients died within 7 months of initial diagnosis, with 25.5% of all deaths within a year not related to the underlying malignancy or non-relapse mortality rate (NRM) (183). These findings are complimented by a study on cGVHD by which the NRM rate of 22% within a five-year follow-up period was attributed to progression of cGVHD infections and non-specified factors (184). While these statistics do not include malignancy-related deaths, it can be understood that the dysregulated immune baseline and heighted activation phase play roles in poor prognosis of GVHD. The presence of autoantibodies has also been documented in GVHD, but it is not known if it mediates or is resultant from this condition (185, 186). HLA mismatches, pre-existing malignancies, and conditioning regimens related to a BMT form a complex interplay that disturbs the immune baseline and culminates into GVHD. This yin-yang immune balance may be temporarily restored with steroids, but patient prognoses of further pathologies and poor survival rates underlie the need for more effective therapeutics.

## 7 Immune related adverse events

First approved by the FDA in 2011, immune checkpoint inhibitors (ICIs) represented a major stride in the mechanistic understanding of cancer immunology (187). Khan and Gerber nicely reviewed that ICIs function to target the immune checkpoint molecules of CTLA-4 and PD-1/PD-L1, which normally work to prevent T cell response to self-antigens and ensure peripheral tolerance is maintained (41). Cancer cells take advantage of these proteins to downregulate T-cell mediated responses against tumors. The therapeutic intervention of ICIs prevents T-cell engagement with checkpoint inhibitors and immunosuppressive signaling, allowing the cytotoxic T cells to continue to expand, secrete cytokines, and perform effector functions against cancer (42, 188). The blockage of checkpoint inhibitors can lead to a variety of damaging and autoimmune-like manifestations such as diabetes, myasthenia gravis, colitis, and rheumatic autoimmune conditions (189, 190). Favoring T-cell activation through ICIs interferes with the yin-yang balance of the immune system, allowing for the immunopathology of hyperinflammation and multiorgan and tissue damage. These manifestations represent a bias towards the yang component seen in **Figure 1**. Termed as immune-related adverse events (irAEs), these manifestations are an extreme side effect of ICIs. The ability of ICIs to prevent downregulation of T cell responses relates to its anticancer effects but is also directly responsible for irAEs. A detailed review of the various body systems associated with irAEs manifestations, as well as suggested therapeutic interventions, has been discussed elsewhere (43). As indicated in **Table 1**, the term irAEs is unique from previously described cytokine storm syndromes (CSS) because it is a catch-all for a slew of symptoms that can result from ICI usage rather than a specific syndrome with well-defined pathology (191–193). General symptoms of irAEs include fever, rash, fatigue, abdominal pain, and diarrhea. Like DRESS, malignancy-associated sHLH, and GVHD, this condition is also initiated by a therapeutic regimen intended to treat an underlying malignancy. One of the most definitive features of cancer is rapid and uncontrollable cell growth (194), representing an immune baseline that contrasts with the yin-yang cycle of cell growth and death that is normally maintained and depicted in **Figure 1**. Regarding immune checkpoint inhibitor (ICI) usage, multiple studies have depicted a bias towards an adult population. Two clinical studies on ICI usage found the median patient age to be over 50 years old (195, 196). Although other studies have enrolled patients as young as 18 to geriatric patients over 80 years old (197, 198), these studies demonstrate a bias towards an adult population. In comparison, the amount of literature detailing ICI usage and long-term outcomes in pediatric patients is sparse. Wong and colleagues suggest that the pronounced gap of ICIs in pediatric populations and subsequent development of irAEs may be due to conflicting reports on efficacy and differences in tumor biology (199). There is hesitation with ubiquitous application of ICIs in pediatric tumors due to low mutational burden that prevents neo-epitopes for T cells and absence of PD-L1 targeted by ICIs (200–203). As there is yet to exist a direct comparison of the frequency of irAEs in adult and pediatric populations, the possibility of an age-dependent presentation requires further elucidation. The combined effects of pre-existing malignancies and median therapeutic age may be associated with the hyperinflammatory response initiated by T cells, leading to the development of a cytokine storm and irAEs in adult patients.

Cytokines play a unique role in irAEs, demonstrated by their involvement with the specific cytokine release syndrome (CRS)-related immune related adverse events (irAEs) and a variety of other irAEs organ manifestations. In a query of 80,700 cases of irAEs from VigiBase, one report analyzed that 55 out of 58 confirmed cases of CRS-irAE lead to serious complications and the final outcome of 20 such cases were not available (204). Although reports of CRS-irAEs are relatively small in comparison to the totality of irAEs, its presentation can serve as a harbinger of both immediate and prolonged immune dysregulation. The shared cytokine profile amongst all irAEs is portrayed through the cytokine-targeted therapies that focus on elevated levels of TNF-α, IL-1, IL-6, IL-12, IL-17, and IL-23 (205). In a study of gastrointestinal cancer patients, it was observed that colitis-irAEs could be correlated with elevated serum levels of IL-6, IL-22, and SCF (206). Elevations of IL-6 and IL-17 have been discussed as well (207, 208) but there is no universal consensus for a cytokine profile for irAEs. The conflicting reports on cytokine elevations in irAEs could be the result of the diversity in manifestations and differences in measurements across different studies. Nevertheless, the field acknowledges that irAEs stem from the hyperactive immune response initiated by immune checkpoint inhibitors (ICIs). The first-line therapeutic for irAEs relies on broad application of corticosteroids (209, 210) resembling the regimen described in previous CSS. With more severe manifestations or presentation of a steroid refractory patient, anti-cytokine therapies such as the TNF-α inhibitor infliximab or IL-6 inhibitor tocilizumab have also been utilized (211, 212). Other irAEs-specific recommendations include hormone replacement therapy for thyroid dysfunction or rituximab to treat neurological and rheumatic manifestations (213–217). Although the presentation of low-grade irAEs has shown to be efficacious in improving anti-cancer responses and overall survival rate of patients (218, 219) the immune landscape may be altered for the worst. It has been estimated that approximately 40% of all ICI patients will develop a chronic irAE that persists at least three months after initiation of therapy, usually related to autoimmune endocrine disorders or rheumatic dysfunction (43, 190, 220, 221). For these patients, the yin phase of the immune response is unable to complement the activation response. Due to pre-existing cancer, the disturbed immune baseline of ICI patients is more responsive to immune-targeted therapeutic regimens. The prolonged activation phase in irAEs is a hyperinflammatory response that is not effectively addressed nor corrected with current therapeutic guidelines.

## 8 Cytokine release syndrome and immune effector cell associated neurotoxicity syndrome

Chimeric antigen receptor T (CAR T) cell therapy has ushered in a new age in the evolving landscape of cancer immunotherapy. This therapy was first approved to treat pediatric and young adult relapsed/refractory B-cell acute lymphoblastic leukemia (R/R B-cell ALL) (27) and has presently expanded to treat other hematological cancers. CAR T-cell therapy harnesses the community’s increased understanding and subsequent manipulation of cancer immunobiology to provide targeted and efficacious treatment options for more patients. The general process of CAR T-cell therapy involves reengineering the patient’s own T lymphocytes with a receptor that recognizes cell surface markers found on B cells and in their malignant transformation, such as CD19 and BCMA (28, 29). At least two prior lines of therapy must fail before becoming eligible for CAR T therapy, seen to be more rigid in multiple myeloma where four prior lines of therapy must be unsuccessful (30, 222). Pre-existing malignancy and multiple lines of failed therapy cause heightened stress in the body, tipping the yin-yang immune response towards hyperinflammation.

CAR T-cell therapy clinical trials of the past and present are wary of cytokine release syndrome (CRS) and accompanying neurotoxic manifestations or immune cell-associated neurotoxicity syndrome (ICANS) that can result (223–226) as has been illustrated in **Figure 3**. Manifestations are graded on a scale of 1 to 4 based upon increasing severity of presentations that can include fever, malaise, headache, dyspnea, and hypotension for CRS and aphasia, altered levels of consciousness, decline in motor control, tremors and seizures, and fatal cerebral edema for ICANS (227, 228). As with ICIs, targeted activation of T lymphocytes is the basis for immunopathology of CRS/ICANS. CAR T cells release cytokines upon engaging with the target antigen that cause both expansion of self and bystander activation of other immune cells, such as monocytes and macrophages (221). A positive feedback loop of cytokine secretion is initiated amongst the cells that leads to cytokine storm and endothelial cell damage. Neurotoxic effects are believed to arise from cytokine storm as well, with leakage of immune cells across a compromised blood-brain barrier directly or indirectly killing neurons (229). Cytokine release represents a double-edged sword in CAR T as it is necessary for their expansion but causes bystander immune cell activation and subsequent organ tissue.

Both the mild and severe clinical presentations of cytokine release syndrome/immune effector cell-associated neurotoxicity syndrome (CRS/ICANS) represent the yang component of the immune response. The main symptomatology of CRS/ICANS has also been touched upon in **Table 1**. Although other adverse events have been reported with CAR T-cell therapy usage (230), the amount of literature on CRS/ICANS and its direct relationship with a cytokine storm make these manifestations most appropriate for consideration in this review. A comparison of adult and pediatric clinical trials leaves room for consideration of age-based severity of ICANS manifestations. The JULIET trial for adult R/R B-cell ALL reported a 12% incidence of grades 3 and 4 ICANS, noting that glucocorticoids successfully resolved most cases (224). This data aligns with the recent UNIVERSAL trial for multiple myeloma in adult patients by which 12% of all patients had potential low grade neurological manifestations that were also resolved with steroid usage (226). In comparison, the ELIANA trial for pediatric and young adult R/R B-cell ALL found that 40% of all patients experienced some degree of neurological manifestations within 8 weeks of initiating therapy (231). While the 13% incidence of grade 3 ICANS mirrors that in the JULIET trial, this pediatric cohort is most noteworthy for the three cases of grade 3 ICANS events were still unresolved at time of therapy cessation or death. The persistence of neurological manifestations represents a disturbed immune baseline in certain pediatric patients that becomes hyperactive with CAR T-cell therapy. Age differences in the immune landscape may then contribute to a baseline that is prone to activation. It must be acknowledged that this perceived difference is not agreed upon by all, as Shalabi and colleagues assert that the incidence of ICANS was found to be similar in both adult and pediatric patients (232). As such, the possible disparity in ICANS incidence and severity deserves further research and consideration on how age can play a role in dysregulated immune responses. Similar to septic shock and HLH conditions, cytokine storm has central role in mediating inflammation in CRS/ICANS.

There is much interest in characterizing a cytokine profile that is predictive of both onset and severity of cytokine release syndrome/immune effector cell-associated neurotoxicity syndrome (CRS/ICANS). Elevated IL-6, IL-8, IL-10, and IFN-γ has been frequently discussed, with a majority of focus placed on IL-6 (28, 225, 226). A meta-analysis of CSF taken from ICANS patients also identified elevated IL-15, IL-10, GM-CSF, IL-2, IL-1RA, and CXCL10 (233). The identification of cytokines elevated in CRS/ICANS shows how a hyperinflammatory immune baseline in cancer patients allows for the development of a cytokine storm. Therapeutic regimens are not unique for this cytokine storm syndrome (CSS), concurrently using corticosteroids and tocilizumab as the first-line therapy (234, 235). Anakinra has also been discussed as both a prophylactic and reactive treatment (236–239). But as with previously mentioned cytokine storm syndromes (CSS), alterative treatment options have yet to replace steroids as the first-line therapeutic for CSS manifestations. The prevalence of steroid treatment is best understood through its mechanism of interfering with the transcription of pro-inflammatory genes, leading to a reduction in cytokine release and associated mediators that are needed for inflammatory processes to occur (240). Although broad enough to be applied to a variety of hyperinflammatory conditions, steroid usage has been linked to many adverse effects including gastrointestinal issues, hypertension, osteoporosis, and the development of cataracts and glaucoma (241, 242). Steroid-induced immunosuppression can dampen the hyperinflammation caused by a cytokine storm, but usage leaves patients vulnerable to both opportunistic pathogens and the risk of future clinical manifestations. There have even been case reports of delayed ICANS that occurred weeks to months after cessation of CAR T-cell therapy (243, 244). Once more, the yin-yang immune response of these individuals becomes fixed in a state of activation and unable to contract to the yin phase and return to a status quo. Usage of targeted therapies may be promising for improved outcomes, but there is yet to exist a therapeutic that is able to correct the inherent immune dysregulation that is present in different types of CSS.

## 9 Discussion and perspectives

The balance in the yin-yang immune response ensures that the powerful and potentially damaging effects of the immune system are constantly kept in check. This occurs through the cyclic activation and contraction phases that both allows for protective inflammatory processes to occur and subsequent downregulation once the threat has been resolved, ensuring the activation phase does not persist past what is beneficial to the host. Cytokine storm syndromes (CSS) represent a dysregulated yin-yang immune response that is predisposed towards an inflammatory or hyperactive immune baseline. The yang component of CSS patients is overactive and creates a hyperinflammatory environment that is not complemented by the contraction phase or yin component. Given the rarity and unique circumstances surrounding CSS, there is an interplay of factors that must be present for a hyperinflammatory response to occur. Age, genetics, hematological and cancerous malignancies, and pre-existing illnesses have varying degrees of involvement in nurturing a dysregulated immune response that is more receptive towards the activation phase. Once these individuals have a trigger, such as illness, pathogen, or therapeutic intervention, they become subject to cellular dysfunctions, multiorgan damage, and systemic inflammation that is representative of cytokine storm.

There has been limited success in counteracting this state of hyperinflammation to allow patients to return to a balanced yin-yang immune response. Steroids are universally applied as a first-line therapeutic for CSS but are wholly unsuccessful in long-term resolution of symptoms, instead causing a variety of undesirable side effects. Alternative treatment options have been used in place of steroids but have yet to be widely recommended or considered. Further development of immunosuppressive and autoimmune states suggests that the immune response is just as dysregulated as before CSS onset. Given the similarities in clinical manifestations, perfunctory yet inefficient use of steroids, and overall poor prognoses of CSS, it is evident that there is an unmet need for more targeted therapies. Given the similarities of the immunopathology and elevated pro-inflammatory cytokines in all conditions, the usage of cytokine-targeted therapies could be an alternative for a more focused treatment approach. By identifying and targeting the cytokine profile for individual conditions, this approach focuses on specific components of the disease and is less subject to unnecessary side effects from non-specific steroid usage. While cytokine-targeted therapies may not be the only solution, effective therapies focusing on the mechanism and immune background of disease onset should be considered. Recognizing the familiar patterns in CSS and affiliated conditions will be central to understanding the mechanisms underlying each disease pathology, trajectory of the immune response, and identifying targeted therapies that allow for restoration of a balance yin-yang immune response and improving the outcome.
