# Corrigendum: Into the storm: the imbalance in the yin-yang immune response as the commonality of cytokine storm syndromes

**DOI:** 10.3389/fimmu.2024.1494424

**Published:** 2024-10-01

**Authors:** Amy Armstrong, Yuting Tang, Neelam Mukherjee, Nu Zhang, Gang Huang

**Affiliations:** ^1^ Department of Cell Systems and Anatomy, Long School of Medicine, University of Texas Health Science Center at San Antonio, San Antonio, TX, United States; ^2^ Department of Microbiology, Immunology, and Molecular Genetics, Long School of Medicine, University of Texas Health Science Center at San Antonio, San Antonio, TX, United States; ^3^ Division of Experimental Hematology and Cancer Biology, Cincinnati Children’s Hospital Medical Center, Cincinnati, OH, United States; ^4^ Department of Urology, Long School of Medicine, University of Texas Health Science Center at San Antonio, San Antonio, TX, United States; ^5^ Department of Pathology & Laboratory Medicine, Long School of Medicine, University of Texas Health Science Center at San Antonio, San Antonio, TX, United States

**Keywords:** cytokine storm syndromes, cytokines, hyperinflammation, immune response, yin-yang

In the published article, there was an error. On page 6, we incorrectly referenced a figure that does not exist within the context of this article, referring to “Figure 32B”.

A correction has been made to **Sepsis and septic shock**. This sentence previously stated:

“From this point, a pathogenic trigger initiates a state of immune activation, leading to a cytokine storm and clinical manifestations of sepsis and septic shock (Figure 32B).”

The corrected sentence appears below:

“From this point, a pathogenic trigger initiates a state of immune activation, leading to a cytokine storm and clinical manifestations of sepsis and septic shock (Figure 3).”

The authors apologize for this error and state that this does not change the scientific conclusions of the article in any way. The original article has been updated.

